# *Toxoplasma gondii* infection in interstate truck drivers: a case–control seroprevalence study

**DOI:** 10.1186/s13071-015-0690-z

**Published:** 2015-02-05

**Authors:** Cosme Alvarado-Esquivel, Sandy Janet Pacheco-Vega, Jesús Hernández-Tinoco, Misael Salcedo-Jáquez, Luis Francisco Sánchez-Anguiano, Luis Omar Berumen-Segovia, Elizabeth Rábago-Sánchez, Oliver Liesenfeld

**Affiliations:** Faculty of Medicine and Nutrition, Juárez University of Durango State, Avenida Universidad S/N, 34000 Durango, Dgo Mexico; Institute for Scientific Research “Dr. Roberto Rivera Damm”, Juárez University of Durango State, Avenida Universidad S/N, 34000 Durango, Durango Mexico; General Hospital, Secretary of Health, Avenida 5 de febrero 220, 34000 Durango, Mexico; Institute for Microbiology and Hygiene, Campus Benjamin Franklin, Charité Medical School, Hindenburgdamm 27, D-12203 Berlin, Germany; Current address: Medical and Scientific Affairs, Roche Molecular Systems, Pleasanton, CA 94588 USA

**Keywords:** *Toxoplasma gondii*, Seroprevalence, Truck drivers, Case–control study, Risk factors

## Abstract

**Background:**

Infection with *Toxoplasma gondii* can be acquired via the ingestion of undercooked or raw meat containing tissue cysts, or via ingestion of water contaminated with oocysts. Professional long distance truck driving may have epidemiological importance for food-borne infections since drivers eat out of home and in places where hygiene and cooking practices are uncertain. We aimed to determine whether interstate truck drivers in Durango, Mexico have an increased risk of infection with *T. gondii* as indicated by seropositivity; and to determine the socio-demographic, work, clinical, and behavioral characteristics associated with *T. gondii* seropositivity in interstate truck drivers.

**Methods:**

Through a case–control study design, 192 truck drivers and 192 controls from the general population of the same region matched by gender and age were examined with enzyme-linked immunoassays for the presence of anti-*Toxoplasma* IgG and IgM antibodies. Socio-demographic, work, clinical and behavioral characteristics from the truck drivers were obtained.

**Results:**

Anti-*T. gondii* IgG antibodies were found in 23 (12.0%) of 192 truck drivers and in 13 (6.8%) of 192 controls (OR = 21.0; 95% CI: 1.23-358.38; *P* = 0.002). Anti-*T. gondii* IgM antibodies were found in 7 (3.6%) cases and in 7 (3.6%) controls (*P* = 1.00). The seroprevalence of *T. gondii* infection was higher in drivers with reflex impairment than in those without this impairment (4/13, 30.8% vs 19/179, 10.6%, respectively; *P* = 0.05), and in drivers with hearing impairment than in those without this impairment (3/7, 42.9% vs 20/185, 10.8%, respectively; *P* = 0.03). Multivariate analysis of work and behavioral characteristics of truck drives showed positive associations of *T. gondii* exposure with trips to the south of Mexico (OR = 3.11; 95% CI: 1.02-9.44; *P* = 0.04) and consumption of horse meat (OR = 5.18; 95% CI: 1.62-16.55; *P* = 0.005).

**Conclusions:**

Results suggest that interstate truck drivers may have an increased risk for *T. gondii* infection, and that *T. gondii* exposure may be impacting neurological functions in truck drivers. Contributing factors for *T. gondii* exposure should be taken into account for the design of optimal prevention measures against *T. gondii* infection.

## Background

The protozoan parasite *Toxoplasma gondii* (*T. gondii*) causes infections in humans all around the world [1]. Most infections with *T. gondii* are acquired by ingesting food or water contaminated with *T. gondii* oocysts shed by cats or by eating undercooked or raw meat containing *T. gondii* tissue cysts [2]. Infections with *T. gondii* are usually asymptomatic but in some individuals infections may lead to severe disease. Toxoplasmosis is characterized by affection of the host’s lymph nodes, eyes, central nervous system, and other organs [2-5]. Infections with *T. gondii* have been associated with schizophrenia [6,7], traffic accidents [8], work accidents [9], and suicide attempts [10,11].

Interstate truck drivers are a group of workers driving trucks for long distances from one state to the other within a country and some of them drive trucks abroad too. This condition may have epidemiological importance for food-borne infectious diseases because such workers eat out of home in places where hygiene and cooking practices are uncertain. Eating away of home has been associated with *T. gondii* exposure in Mexico [12]. In a previous study in patients with liver disease, we observed that patients with an occupation of truck driver had a higher seroprevalence of *T. gondii* infection than those without this occupation [13]. However, case–control studies to assess the association of *T. gondii* exposure and interstate truck driver occupation have not been performed. Therefore, we performed an age- and gender matched case–control study to determine the association of *T. gondii* seropositivity with interstate truck driver occupation in Durango, Mexico. Furthermore, we investigated the socio-demographic, work, clinical, and behavioral characteristics associated with *T. gondii* seropositivity in the interstate truck drivers studied.

## Methods

### Study design and study populations

Through a case–control study design, we studied the association of seropositivity to *T. gondii* with interstate truck drivers. The present study was performed in Durango City, Mexico from November 2013 to July 2014.

*Interstate truck drivers*: One hundred and ninety two interstate truck drivers were enrolled in the study. Inclusion criteria for the cases were current working as interstate truck drivers for at least one year, aged 18 years and older, any gender, and who accepted to participate in the study. As a strategy to enroll drivers, they were invited to participate in gas stations, small motels for truck drivers, and truck stops on the roads. One hundred and eighty seven of them were males and five were female. The mean age of the drivers was 43.40 ± 10.16 years (range: 20–71 years).

*Control subjects*: One hundred and ninety two control subjects without truck driver occupation matched with drivers by age and gender were included in the study. Control subjects were randomly selected from the general population of Durango City, Mexico. The mean age in controls was 43.45 ± 10.21 (range: 20–71 years) and comparable with that in truck drivers (*P* = 0.96).

### Socio-demographic, clinical, work and behavioral data

We obtained the socio-demographic, work, clinical and behavioral characteristics of the participants with the aid of a standardized questionnaire. Socio-demographic items included age, gender, birthplace, residence area, educational level, and socio-economic level. Work characteristics assessed in truck drivers included number of years in the activity, type of materials transported, frequency and duration of trips, regions and number of states visited within Mexico, work abroad, traffic accidents, type of places of getting meals out of home, transportation of animals, and washing the truck by themselves. Clinical data in truck drivers included presence of any disease, presence or history of lymphadenopathy, frequent headache, dizziness, impairments in memory, reflexes, hearing and vision, and history of blood transfusion, transplant or surgery. Behavioral items included contact with animals or cat feces, type of meat consumed, consumption of raw or undercooked meat, unpasteurized milk, dried or cured meat, consumption of unwashed raw vegetables, fruits, or untreated water, frequency of eating away from home (in restaurants or fast food outlets), washing hands before eating, contact with soil, and type of flooring at home.

### Laboratory tests

A blood sample from each participant was obtained. Blood samples were centrifuged and sera were obtained and kept frozen at −20°C until analyzed. Sera were analyzed by qualitative and quantitative methods for anti-*T. gondii* IgG antibodies with the commercially available enzyme immunoassay kit “*Toxoplasma* IgG” (Diagnostic Automation Inc., Calabasas, CA, USA). Anti-*T. gondii* IgG antibody levels were expressed as International Units (IU)/ml, and a result equal to or greater than 8 IU/ml was considered positive. In addition, sera positive for anti-*T. gondii* IgG antibodies were further analyzed for anti-*T. gondii* IgM antibodies by the commercially available enzyme immunoassay “*Toxoplasma* IgM” kit (Diagnostic Automation Inc., Calabasas, CA, USA). All tests were performed following the instructions of the manufacturer.

### Statistical analysis

We performed the statistical analysis with the aid of Epi Info version 7 and SPSS 15.0 (SPSS Inc. Chicago, Illinois). For calculation of the sample size, we used a 95% confidence level, a power of 80%, a 1:1 proportion of cases and controls, a reference seroprevalence of 6.1% [14] as the expected frequency of exposure in controls, and an odds ratio of 2.8. The result of the sample size calculation was 175 cases and 175 controls. Age among the groups was compared by the student’s *t* test. For comparison of the seroprevalences of *T. gondii* infection between truck drivers and control subjects, the McNemar’s test was used. We used the Pearson’s chi-squared test and the two-tailed Fisher’s exact test (when values were less than 5) to determine the association of *T. gondii* seropositivity and the characteristics of the drivers studied. Odds ratio (OR) and 95% confidence interval (CI) were calculated by multivariate analysis using logistic regression with the Enter method. Variables were included in the multivariate analysis if they had a *P* value less than 0.05 in the bivariate analysis. The Hosmer-Lemeshow goodness of fit test was used to assess the fitness of our regression model. Statistical significance was set at a *P* value less than 0.05.

### Ethical aspects

This study was approved by the Institutional Ethical Committee of the General Hospital of the Secretary of Health in Durango City. The purpose and procedures of the study were explained to all participants, and a written informed consent was obtained from each participant. Participants did not receive financial compensation.

## Results

Anti-*T. gondii* IgG antibodies were found in 23 (12.0%) of 192 interstate truck drivers and in 13 (6.8%) of 192 controls. The difference between the seroprevalences in cases and controls was statistically significant according to the McNemar’s pair test (OR = 21.0; 95% CI: 1.23-358.38; *P* = 0.002). Of the 23 anti-*T. gondii* IgG positive drivers, 11 (5.7%) had IgG levels higher than 150 IU/ml, 1 (0.5%) between 100 to 150 IU/ml, and 11 (5.7%) between 8 to 99 IU/ml. In contrast, of the 13 anti-*T. gondii* IgG positive controls, 8 (4.2%) had IgG levels higher than 150 IU/ml, 1 (0.5%) between 100 to 150 IU/ml, and 4 (2.1%) between 8 to 99 IU/ml. Prevalence of high (>150 IU/ml) anti-*T. gondii* IgG levels was comparable among drivers and controls (*P* = 0.48). Anti-*T. gondii* IgM antibodies were found in 7 (3.6%) cases and in 7 (3.6%) controls (*P* = 1.00).

The socio-demographic characteristics among *Toxoplasma*-positive and *Toxoplasma*-negative drivers were not significantly different (Table 1). Of the work characteristics, only two variables showed potential association with seropositivity by bivariate analysis: “trips to the south of Mexico” (*P* = 0.007) and “number of eating places during trips” (*P* = 0.01). Concerning clinical characteristics of drivers, the seroprevalence of *T. gondii* infection was higher in drivers with reflex impairment than in those without this impairment (4/13, 30.8% vs 19/179, 10.6%, respectively; *P* = 0.05), and in drivers with hearing impairment than in those without this impairment (3/7, 42.9% vs 20/185, 10.8%, respectively; *P* = 0.03). In the subset of 62 drivers with traffic accidents, the frequency of *T. gondii* infection was significantly higher in drivers with reflexes impairment than in those without this impairment (3/5, 60% vs 7/57, 12.3%, respectively; *P* = 0.02). In the same subset of drivers with traffic accidents, the frequency of *T. gondii* infection was similar in drivers with hearing impairment than in those without this impairment (0/1, 0% vs 10/61, 16.4%, respectively; *P* = 1.0). Other clinical characteristics assessed were not associated with *T. gondii* seropositivity.

**Table 1:** **Socio-demographic characteristics of truck drivers and seroprevalence of** ***T. gondii*** **infection**

		**Prevalence of**		
	**Subjects tested**	***T. gondii*** **infection**		***P value***
**Characteristic**	**No.**	**No.**	**%**	
Age groups (years)				
30 or less	21	1	4.8	0.25
31-50	120	13	10.8	
>50	51	9	17.6	
Sex				
Male	187	22	11.8	0.47
Female	5	1	20.0	
Birth place				
Durango State	104	9	8.7	0.12
Other Mexican state	88	14	15.9	
Residence				
Durango State	116	11	9.5	0.18
Other Mexican state	76	12	15.8	
Residence area				
Urban	149	20	13.4	0.41
Suburban	36	2	5.6	
Rural	7	1	14.3	
Educational level				
No education	2	1	50.0	0.36
1-6 years	71	7	9.9	
6-12 years	113	14	12.4	
>12 years	6	1	16.7	
Socio-economic level				
Low	38	7	18.4	0.37
Medium	153	16	10.5	
High	1	0	0.0	

Of the behavioral characteristics, only seven variables showed potential association with *T. gondii* seropositivity by bivariate analysis (*P* values <0.05): consumption of meat from goat, rabbit, horse, armadillo, iguana, snake and type of flooring at home. Multivariate analysis of work and behavioral characteristics of truck drives with *P* value <0.05 obtained in bivariate analysis showed two positive associations of *T. gondii* exposure including trips to the south of Mexico (OR = 3.11; 95% CI: 1.02-9.44; *P* = 0.04) and consumption of horsemeat (OR = 5.18; 95% CI: 1.62-16.55; *P* = 0.005) (Table 2). The result of the Hosmer-Lemeshow test (*P* = 0.47) indicated an acceptable fit of our regression model.

**Table 2:** **Multivariate analysis of selected characteristics of truck drivers and their association with** ***T. gondii*** **infection**

**Characteristic**	**Odds ratio**	**95% confidence interval**	***P value***
Trips to Southern Mexico	3.11	1.02-9.44	0.04
Number of eating places	2.39	0.84-6.76	0.10
Consumption of meat from:			
Goat	2.51	0.71-8.84	0.14
Rabbit	1.21	0.39-3.76	0.73
Horse	5.18	1.62-16.55	0.005
Armadillo	2.60	0.46-14.49	0.27
Iguana	1.59	0.50-5.10	0.42
Snake	1.98	0.68-5.76	0.20
Soil floor at home	1.83	0.67-4.99	0.23

## Discussion

Toxoplasmosis is a food-borne [15] and a water-borne disease [16]. The seroprevalence of infection with *T. gondii* differs from state to state or region in the general population dependent on the presence or absence of risk factors for the infection. People living in low seroprevalence areas therefore have an increased risk for infection when visiting high seroprevalence states. The frequent traveling to different regions could therefore represent a risk factor for infection with *T. gondii* in interstate truck drivers. In the present study, the seroprevalence of *T. gondii* infection (12%) was significantly higher in interstate truck drivers than in control subjects of the general population of Durango City. This two-fold increase points towards an association of *T. gondii* infection with an occupation of interstate truck driver. To the best of our knowledge, this is the first age- and gender matched case control study that demonstrates this association.

We attempted to identify contributing factors for infection in the truck drivers. The number of variables assessed in the present study was large. However, the number of variables was reduced by performing firstly bivariate analysis of all variables followed by multivariate analysis of a small number of selected variables with potential association (*P* < 0.05) with *T. gondii* seropositivity. None of the socio-demographic characteristics of the drivers was associated with *T. gondii* seropositivity. Multivariate analysis of work and behavioral characteristics of drivers showed that *T. gondii* infection was associated with work trips to the south of Mexico and with consumption of horsemeat. The seroprevalence of *T. gondii* infection in the general population is higher in southern Mexican states than in northern Mexican states. Seroprevalences higher than 50% have been found in southern Mexican states [17]. In contrast, a 6.1% seroprevalence of *T. gondii* infection in the general population in the northern Mexican city of Durango was found [14]. Differences in climatic conditions may contribute to a higher seroprevalence of *T. gondii* infection in southern Mexican states. Seroprevalence of *T. gondii* infection is higher in humid than in dry climates [18-20]. Durango has a dry climate while southern Mexican states have a more humid climate. Regarding the association of *T. gondii* infection with consumption of horsemeat, this is the first time we found such association in a Mexican population. Our result strengthens the epidemiological role of horsemeat as a source of *T. gondii* infection. A 6.1% seroprevalence of *T. gondii* infection in horses in Durango State was reported recently [21]. We are not aware of further studies on the seroprevalence of *T. gondii* infection in equids in other Mexican states. Viable *T. gondii* has been recovered from tissues of equids experimentally infected with parasite oocysts [22]. In addition, several cases of clinical toxoplasmosis due to consumption of horsemeat have been described [23,24]. None of the drivers studied referred to eat raw meat. Nevertheless, there is no certainty that tissue cysts, if present in the horsemeat consumed, were all inactivated by cooking. Several factors including temperature and time of cooking, and thickness of meat should be consider for inactivation of tissue cysts. In a study using infectivity bioassays in cats and mice, inactivation of tissue cysts in sheep meat was reached by cooking meat at 60°C or 100°C for 10 minutes [25]. Further research to determine the heating parameters for inactivation of tissue cysts in horsemeat is needed.

Infection with *T. gondii* has been associated with traffic accidents [8] and reflex impairment [26]. In the present study, drivers with traffic accidents had a higher, but not statistically significant, seroprevalence of *T. gondii* infection than drivers without traffic accidents. The lack of association of *T. gondii* infection and traffic accidents may be due to a small sample size of our study population. However, since reflexes impairment showed a borderline (*P* = 0.05) association with *T. gondii* exposure in the present study we further assess whether seropositivity to *T. gondii* in the subset of drivers with traffic accidents differ between drivers with reflexes impairment and those without this impairment. Remarkably, seroprevalence of *T. gondii* infection in the subset of drivers with traffic accidents was significantly higher in drivers with reflex impairment than in those without this impairment. Results thus suggest that infection with *T. gondii* might lead to reflexes impairment that in turn may contribute for traffic accidents. Chronic infection with *T. gondii* has been associated with slowing of neural processing speed [27], reduced psychomotor performance and changes in behavior [28]. In the present study, we also found that *T. gondii* infection was associated with hearing impairment. This impairment might also contribute for traffic accidents. Infection with *T. gondii* has been associated with hearing loss in congenital toxoplasmosis. This hearing loss is likely caused by inflammation induced by tachyzoites of *T. gondii* [29]. Congenital hearing loss has not always been associated with primary infection during pregnancy [30], and very little is known about hearing loss caused by *T. gondii* in adults. In a previous study in an ethnic group (Tepehuanos) in Durango, Mexico, we found a significantly higher seroprevalence of IgM antibodies against *T. gondii* in subjects suffering from hearing impairment than those without this impairment [31]. It is unclear whether infection with *T. gondii* may affect the inner ear. However, dizziness -a symptom that may reflect disease in the inner ear- has been associated with *T. gondii* infection in the ethnic group of Huicholes [32] and in migrant agricultural workers in Durango, Mexico [33]. Further research to elucidate the role of *T. gondii* infection in inner ear disease is needed.

## Conclusions

Results of this first case–control study suggest that interstate truck drivers in Mexico are at increased risk for *T. gondii* infection, and that *T. gondii* exposure may impact the health of truck drivers. Contributing factors for *T. gondii* exposure such as horsemeat should be taken into account for the design of optimal prevention measures against *T. gondii* infection.
